# Musculoskeletal Deficits and Cognitive Impairment: Epidemiological Evidence and Biological Mechanisms

**DOI:** 10.1007/s11914-022-00736-9

**Published:** 2022-06-29

**Authors:** Sophia X. Sui, Julián Balanta-Melo, Julie A. Pasco, Lilian I. Plotkin

**Affiliations:** 1 Epi-Centre for Healthy Ageing, Deakin University, IMPACT - Institute for Mental and Physical Health and Clinical Translation, PO Box 281 (Barwon Health), Geelong, VIC 3220 Australia; 2 Department of Anatomy, Cell Biology & Physiology, Indiana University School of Medicine, 635 Barnhill Drive, MS5022A, Indianapolis, IN 46202 USA; 3 Indiana Center for Musculoskeletal Research, Indiana University School of Medicine, Indianapolis, IN USA; 4 Richard L. Roudebush Veterans Administration Medical Center, Indianapolis, IN USA; 5 Universidad del Valle School of Dentistry, Cali, Colombia; 6 Department of Medicine–Western Campus, The University of Melbourne, St Albans, VIC Australia; 7 Department of Epidemiology and Preventive Medicine, Monash University, Melbourne, VIC Australia; 8 University Hospital Geelong, Barwon Health, Geelong, VIC Australia

**Keywords:** Cognition, Bone, Muscle, Musculoskeletal system, Nervous system

## Abstract

**Purpose of Review:**

Cognitive impairment is associated with obesity, sarcopenia, and osteoporosis. However, no critical appraisal of the literature on the relationship between musculoskeletal deficits and cognitive impairment, focusing on the epidemiological evidence and biological mechanisms, has been published to date. Herein, we critically evaluate the literature published over the past 3 years, emphasizing interesting and important new findings, and provide an outline of future directions that will improve our understanding of the connections between the brain and the musculoskeletal system.

**Recent Findings:**

Recent literature suggests that musculoskeletal deficits and cognitive impairment share pathophysiological pathways and risk factors. Cytokines and hormones affect both the brain and the musculoskeletal system; yet, lack of unified definitions and standards makes it difficult to compare studies.

**Summary:**

Interventions designed to improve musculoskeletal health are plausible means of preventing or slowing cognitive impairment. We highlight several musculoskeletal health interventions that show potential in this regard.

## Introduction

Musculoskeletal and cognitive function declines often occur at the same time, for example with aging or in individuals with dementia [1–3]. Cognition is an intellectual or mental process via which organisms obtain knowledge and cognitive impairment (diminished intellectual and/or mental functioning) is associated with obesity [4]. Further, brain function and body composition are linked [4–7]. In particular, obesity in middle age has been linked with increased risk of developing Alzheimer’s disease (AD) [8, 9]. However, whether cognitive and musculoskeletal abnormalities share common mechanistic bases or they appear at the same time due to common risk factors is not completed clear and is a subject of continuous research.

**Fig. 1:**
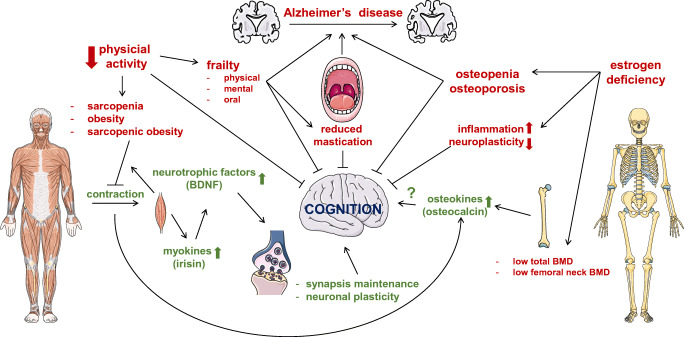
Schematic representation of the nervous-musculoskeletal systems crosstalk. The figure depicts some of the mechanisms by which this crosstalk occurs. The balance among factors produced by the brain, bone, and skeletal muscle is required not only for each tissue homeostasis, but also for the health of other tissues through the production of hormones, cytokines, and mechanical forces. Further research is needed to understand the physiological, cellular, and molecular mechanisms behind the link between the cognition and the musculoskeletal system in health and disease. BDNF, brain derived neurotrophic factor; BMD, bone mineral density

Efforts to establish the mechanistic links between brain and musculoskeletal function have been undertaken. For example, studies showed that metabolically active tissues such as skeletal muscle release neurotrophic factors that regulate synapses in the brain [10]. One of such factors is the brain-derived neurotrophic factor (BDNF), released during skeletal muscle contraction [11], and which absence has been linked to neurodegenerative processes [12]. Similarly, serotonin controls bone mass accrual by acting on its receptor in the receptors on ventromedial hypothalamic neurons [13]. Further, several factors released by osteoblasts and osteocytes in bone, including osteocalcin, sclerostin, and fibroblast growth factor 23, can cross the blood–brain barrier and alter brain function [14]. Herein, we review the current literature on brain-musculoskeletal system interactions (summarized in Figure 1) and propose future directions that might help resolve controversies in the field.

## Musculoskeletal System and Cognition

Mounting evidence suggests that physical activity is connected to cognitive development and brain evolution, whereas lifestyles and habits characterized by prolonged stasis (e.g., office work, sedentary entertainment) are associated with increased risk for cognitive impairment [15, 16]. The effects on musculoskeletal conditions and cognitive status of 2-h prolonged sitting [17] and standing [18] during office work were evaluated in an adult population in Australia. Prolonged sitting and standing were associated with increased whole-body musculoskeletal discomfort based on the Nordic Musculoskeletal Questionnaire [17, 18], with detrimental effects on mental status and attention reaction responses [17, 18]. Contrasting results were found for problem-solving skills, assessed with the Ruff Figural Fluency Test, showing improvements during prolonged sitting, but the opposite effect during prolonged standing [17, 18]. Moreover, prolonged standing combined with a foot movement exercise further decreased problem-solving skills while focused musculoskeletal discomfort to the foot and ankle regions [19]. Other studies suggest that prolonged standing decreases cognitive performance during complex tasks [20]. Therefore, moving from sitting to standing while working, involving minimal physical activity, must be evaluated further to understand the mechanisms behind the relationship between musculoskeletal health and cognitive status.

The effects of intermittent physical activity were assessed in a study involving 8 middle-age men and 3 women, by determining the effect of three different disruptive activities (social interaction, functional resistance training, and walking) on cognitive performance and salivary cortisol levels (as a marker of stress) during 3-h sitting [21]. Using a memory task evaluation and the numerical *n*-back test method, this study found that walking improves cognitive performance. Interestingly, the study also determined a reduction in salivary cortisol levels when participants were exposed to any of the three disruptive activities. The *n*-back test is useful to assess working memory, since it allows the participants to recall the last in a series of events [22]. Another study of healthy adults 65 years and older showed improved performance on memory task when the letter *n*-back test was assessed 15 and 45 min after stationary bicycle exercise [23]. Further, following exercise, activity in the parietal brain region was found higher than that of the frontal region, as determined by brain hemoglobin concentration [23]. Further, a positive relationship between the intensity of daily physical activities (from sedentary to highly active) and bone mass was found in adults aged over 70 years, with sex-dependent differences in certain brain regions [24]. Additionally, the later study revealed that women with bone fracture history had been less active earlier in life than women without fractures [24].

Musculoskeletal and dental conditions affecting mastication are also associated with negative effects on cognition. Thus, studies in Japanese individuals 65 years and older show an association between either having less than 20 functional teeth and reduced occlusal force or being fully edentulous and cognitive impairments [25, 26]. Furthermore, a systematic review identified a study linking reduced cognitive performance (based on the Mini-Mental State Exam (MMSE)) with impaired masticatory function in AD patients compared to an age-matched healthy population [27]. Nonetheless, the relationship between mastication and cognitive impairment is not fully understood [28].

## Skeletal Muscle and Cognition

### Sarcopenia and Cognitive Impairment

Sarcopenia is generally defined as age-related progressive loss of muscle mass and function, although the working definition varies among different groups [29, 30]. Thus, several operational definitions of sarcopenia are in use, including those provided by the European Working Group on Sarcopenia in Older People [31], the United States Foundation for the National Institutes of Health (FNIH) [32], the Asian Working Group for Sarcopenia (AWGS) [33], and the Sarcopenia Definition and Outcomes Consortium [34]. Therefore, the prevalence of sarcopenia reported in the literature varies substantially within and across geographic areas, due to differences in the criteria and cutoff points applied [35, 36]. Researchers conducting the Geelong Osteoporosis Study (GOS) [37] described the consequences of applying different criteria and cutoff points (international and population-specific) in a homogeneous sample for prevalence estimates for the Australian population [38, 39]. The main finding was that, across all the definitions, the prevalence of sarcopenia increased with increasing age; however, the varied criteria and cutoff points resulted in inconsistent case ascertainment. The GOS explored the relationship between several muscle parameters and overall cognitive function, specific cognitive domains, and cognitive impairment among men [40]. The findings suggested that muscle parameters, especially muscle function [41••], muscle quality [42•], and muscle density [43••], are associated with certain cognitive domains (including working memory, attention, and information processing speed) independent of age, physical activity levels, education, and lifestyle factors. However, these associations were not detected for all the cognitive domains tested. Similar results on the association between dynapenia (age-associated muscle strength loss not caused by neurologic or muscular diseases) and low cognition were reported in female GOS participants [44•].

In summary, inconsistencies in studies associating muscle health with cognition could be due to differences in criteria used to define sarcopenia, sarcopenic obesity, and cognitive deficits. Evidence for mechanisms linking muscle health with certain brain functions also remain unclear.

### Sarcopenic Obesity and Cognitive Impairment

Sarcopenic obesity is a condition characterized by concurrent high adiposity levels and low muscle mass and function with advanced age [45]. The association between sarcopenic obesity and cognition was assessed in 353 community-recruited USA participants aged 40 years and over [46]. Body composition was measured by bioelectrical impedance analysis. Obesity was determined by body mass index (BMI) and fat percentage. Global cognition was assessed using the Montreal Cognitive Assessment. Specific cognitive domains in verbal fluency and mental flexibility were assessed using the animal naming test (participants are asked to name as many animals as they could within one minute). Visual search speed, scanning, and processing speed were assessed using Trail Making A. The authors recommended that sarcopenia and sarcopenic obesity should be regarded as clinical indicators of cognitive impairment, listing potential mechanisms that explain sarcopenic obesity and cognitive deficits association, including low-grade chronic inflammation, oxidative stress, and insulin resistance; however, no biomarker data was included in the report.

The association between sarcopenic obesity and cognitive impairment was examined in 948 community-based Chinese participants aged 60 years and over (51% female) [47]. Body composition was measured by bioelectrical impedance analysis and cognition by MMSE. Sarcopenia was defined using the AWGS criteria, and obesity was determined by body fat percentage (fat mass/weight). Six percent of participants were identified as sarcopenic obese. This study reported an independent association between sarcopenic obesity and cognitive impairment and proposed that the mechanisms underlying the link between muscle, fat, and cognition were inflammation, insulin resistance, and decreased growth hormone secretion, but these hypotheses were not tested.

The association between sarcopenic obesity and cognitive performance was also examined in 1235 Singaporean patients (~48% female) aged 45 years and over with type 2 diabetes; all were attending diabetes care [48]. Body composition was assessed using bioelectrical impedance analysis, and the Repeatable Battery for the Assessment of Neuropsychological Status and MMSE were applied for assessing cognition. This study identified an association between sarcopenic obesity and poor cognitive performance, particularly in the domains of memory and language. The authors acknowledged that the mechanisms underlying these dual deficits in brain and body were not fully understood; however, it is likely that in these patients, abnormal levels of insulin affect amyloid β metabolism, which controls neuronal function.

Further, in a longitudinal, population-based study in the USA, the association between sarcopenic obesity and incident cognitive function was determined in 5822 participants (~56% female) aged 65 years old and over, without cognitive impairment at baseline [49]. They adopted the FNIH definition of sarcopenia, and defined obesity by BMI. Cognition was assessed using AD-8 score or immediate/delayed recall, orientation, clock-draw test, or date/person recall. At baseline, 12.9% of subjects were identified as having sarcopenic obesity; 21.2% were identified with cognitive impairment at follow-up. Sarcopenia obesity and sarcopenia alone were significantly associated with higher risk of cognitive impairment. On the other hand, a study including 542 participants 21–90 years recruited from the Chinese community produced contrasting findings [50]. The researchers obtained body composition data using dual-energy x-ray absorptiometry (DXA) and defined obesity as the upper two quintiles of fat mass index. They employed a sarcopenia definition based on the 2019 AWGS criteria. Cognitive impairment was determined using the Repeatable Battery for the Assessment of Neuropsychological Status. This battery included 12 tests to assess immediate memory, visuospatial/constructional abilities, language, attention, and delayed memory. Sarcopenia and sarcopenic obesity were not associated with cognitive impairment, but obesity alone and muscle function (grip strength or gait speed) were. The authors speculated that insulin resistance underpins the association.

## Bone and Cognition

Osteoporosis is characterized by low bone mass, and is defined by the number of standard deviations a patient’s BMD differs from the average of a population of the same age and sex (*Z*-score) or from a young normal reference value (*T*-score). *T*-scores lower than −2.5 standard deviations are indicative of osteoporosis, whereas values between −1.0 and −2.5 lead to a diagnosis of osteopenia, a milder condition [51], as defined by the Word Health Organization [52]. Low BMD increases risk for fracture [53] and has been associated with high morbidity and mortality [54], in particular among older individuals.

Several epidemiologic studies have looked at the potential correlation between low bone mass and cognitive decline. The PRESENT project 2018 [55] examined the association between BMD and cognition in 650 Koreans without stroke or dementia, aged 50 years or older. MMSE was used to assess cognition in 197 participants with osteopenia and 154 with osteoporosis. As expected, osteoporosis was more common among women than among men, but low BMD was associated with cognitive impairment in both genders, although the association appeared stronger in women for both osteopenia and osteoporosis. The authors listed possible common mechanisms underlying this association, including estrogen deficiency. Postmenopausal estrogen deficiency leads to an initial increase in bone formation and resorption, with bone loss resulting from remodeling imbalance. In the brain, estrogen reduces inflammation and promotes neuroplasticity in the brain processes crucial for learning and memory [56].

Similarly, a systematic literature review and meta-analysis reported that both lower BMD and lower femoral neck BMD were linked to increased risk of AD and gender seems to play a role in this association [57]. Further studies, under the Bushehr Elderly Health Program [58], examined gender-specific cross-sectional associations between osteoporosis and cognitive impairment in a community-dwelling sample of 1508 Iranian participants aged 60 years and over (~49% female). BMD was assessed using DXA; osteoporosis and osteopenia were identified by DXA-derived BMD at any skeletal site. Cognition was assessed using Mini-Cog and categorical verbal fluency tests. Five hundred and ninety-eight participants had osteoporosis and 677 had cognitive impairment. Osteoporosis at the spine and total hip was associated with increased risk of cognitive impairment in women and, conversely, cognitive impairment was associated with increased risk of spinal osteopenia/osteoporosis, total hip osteoporosis, and whole-body osteoporosis in women. These associations were not found in men. The authors propose that gender differences identified in this study are due to changes in estrogen levels in women during their lifespan and suggested that hypothalamic–pituitary–adrenal axis dysregulation reflected the dual decline in bone health and cognitive function [58].

The associations between osteoporosis and cognitive function were also examined in 260 hospitalized Korean patients (59% female) recovering from acute stroke [59]. Osteoporosis was defined by *T*-score ≤−2.5 or low BMD in the femoral neck or lumbar spine, and cognitive impairment was assessed by the Korean MMSE. Patients with osteoporosis before and after the recovery phase had higher prevalence of cognitive impairment. In addition, women who experienced significant cognitive decline in the first 5 years after stroke had increased risk of fracture over the next decade. None of the associations identified in women was found in men. The authors recommended further investigation of gender-specific biological mechanisms that might underlie these associations.

Using the Canadian Multicentre Osteoporosis Study (CaMos), the relationship between cognitive decline, bone loss, and fracture risk was examined in 2361 participants (74% female) selected from the general population [60]. Cognition was assessed using the MMSE. In women, but not in men, there was an association between cognitive decline and bone loss. The authors suggested that estrogen could mediate this association [60]. The association between dementia, low BMD, and osteoporosis was examined in 363 Turkish adults aged 65 years and over (63% female) [61]. In this study, BMD assessed by DXA was found lower in participants with dementia, but without differences based on the type of dementia (AD, vascular dementia, or mixed dementia), gender, disease duration, or severity.

In summary, cross-sectional and longitudinal data support an association between osteoporosis and poor cognition, independent of aging. This pattern appears to be more evident in women than in men, although further research is needed to identify mechanisms linking these deficits in bone and brain.

## Muscle and Bone Crosstalk and Cognitive Impairment

A recent report examined lean mass (a surrogate for skeletal muscle mass) and bone mass in association with cognitive status among 535 Taiwanese participants, aged 65 years or over, of which 67.3% had normal cognition status, 18.3% had mild cognitive impairment, and 14.4% had a diagnosis of dementia [62]. An association between bone loss and cognitive impairment was detected. In addition, the authors claimed that diminishing lean mass reduced BMD, so was an indirect contributor to cognitive impairment. Parallel losses of lean and bone mass have also been reported using data from the GOS [63].

## Frailty and Cognitive Impairment

Frailty is reduced resilience to stressors that may lead to declines in multiple functional systems. It is associated with skeletal muscle weakness, fatigue, reduced mobility, low physical activity, and weight loss [64]. Cognitive frailty is defined as concurrent physical frailty and cognitive impairment (excluding dementia and AD) [65], and is associated with adverse health outcomes, such as functional disability, depression, malnutrition, hospitalization, impaired quality of life, loss of independence, and, ultimately, mortality, in the elderly [66–68]. There is currently no universally recognized definition of cognitive frailty, which has been argued to constitute an independent dimension of frailty [69]. Our current understanding of the neuropathological pathways in cognitive frailty is insufficient to develop a cost-effective screening tool, partially because the high cost of specialized equipment (e.g., functional MRI) means that most research involved only small numbers of participants, giving low statistical power for detecting differences and changes. The pathological pathways in cognitive frailty are unclear. As described above, contracting skeletal muscle is a major source of neurotrophic factors, including BDNF, which regulates synapses in the brain [10]. Thus, BDNF is a plausible candidate for the as-yet unidentified mechanism linking skeletal muscle and brain function. Furthermore, skeletal muscle activity has immune and redox effects that support brain function [70] and reduce muscle catabolism [71]. Muscle loss, muscle weakness, fat infiltration into muscle, and frailty, in turn, appear to be associated with systemic and central inflammation and have been linked with impaired synaptic neuroplasticity and cognitive decline [72].

Oral (or dental) frailty has emerged as an indicator on the relationship between oral status and overall health [73]. It is characterized by a reduction in oral activity combined with both musculoskeletal and cognitive impairments. Particularly during aging, musculoskeletal functions such as mastication, swallowing, occlusal force, and tongue pressure are compromised, contributing to declined overall health status and frailty [74, 75••]. Although oral health has been extensively assessed in terms of neurodegeneration risk (e.g., periodontal pathogens and AD), further research on how oral frailty as a musculoskeletal syndrome may promote cognitive impairment is required [74, 75••].

## Molecular and Cellular Mechanisms Underlying Musculoskeletal Deficits and Cognitive Impairment

As recent reviews [76–78] show, skeletal muscle and bone are dynamic tissues able to communicate both biomechanically and molecularly. In addition, molecular factors released from skeletal muscle and bone (myokines and osteokines, respectively) affect cognitive processes [76–78]. Since myokines and osteokines may be released in response to physical activity, it is reasonable to consider cellular and molecular crosstalk as potential mechanisms underpinning the relationship between the musculoskeletal system and cognition. Myokines, osteokines, and sex hormones are being studied in the hope of revealing avenues for development of therapies for cognitive detriments caused or aggravated by musculoskeletal deficits.

### Myokines and Cognition

During contraction, skeletal muscle releases molecular factors that may affect cognitive function, such as BDNF, a neurotrophin required in adults for the maintenance of synaptic connections and adaptive neuronal plasticity, regulating cognitive processes such as learning and memory [79]. A study showed that after long-term voluntary exercise, adult male mice exhibited a lactate-dependent increase in hippocampal BDNF [80]. Interestingly, lactate (a metabolite released from muscle during exercise) was responsible for improvement in both learning and memory in these mice, and the induction of the hippocampal BDNF expression was found to be dependent on the sirtuin 1 (SIRT1)/peroxisome proliferator–activated receptor-γ coactivator 1-α (PGC1α)/fibronectin type III domain-containing 5 (FNDC5) pathway [80]. Furthermore, SIRT1 knockout male mice were found to be more anxious than wild-type mice and suffering cognitive impairment characterized by reduced learning abilities and memory [81]. In a rat model of AD, direct intervention in the hippocampus with the 42 amino acid form of amyloid β (Aβ1–42) resulted in cognitive impairment by suppressing PGC1α/FNDC5/BDNF signaling [82]. However, the cognitive impairment was partially reversed by moderate physical activity, revealing a recovered PGC1α/ FNDC5/BDNF pathway [82].

In addition, BDNF is released in response to muscle contraction [83], and percutaneous electrical stimulation of the hindlimb muscles in a rat model of spinal cord injury significantly increased BDNF levels in both the anterior tibialis and the vertebral column [84]. Importantly, deletion of BDNF in skeletal muscle in mice resulted in a fatigue-resistant muscle phenotype, migrating from fast to slow muscle fibers in glycolytic muscles tibialis anterior and extensor digitorum longus [85]. In contrast, BDNF overexpression increased the glycolytic and fast fiber phenotype of the muscles [85]. This is consistent with clinical findings, since BDNF levels in skeletal muscles induced by controlled physical activity were found to be correlated positively with muscle phenotypic changes favoring type II muscle fibers (fast and glycolytic) [86]. Moreover, serum BDNF was increased in sedentary subjects 1 h after training, but this was not found in trained young and adult patients, suggesting the relevance of physical conditioning when assessing the effect of training on BDNF induction [86]. In addition, the decrease of BDNF after training was correlated with improvement in cognitive processes such as visuospatial and verbal skills (measured using a before-and-after Addenbrooke’s Cognitive Examination-Revised test) [86]. Consistent results have been obtained in experimental models, involving young and aged rats, suggesting a role of the PGC1α/ FNDC5/BDNF pathway in the protection of cognition from a musculoskeletal health approach [87].

BDNF expression can also be affected by altered mastication. In growing mice receiving a soft diet, learning and memory processes were impaired, compared to mice eating standard chow, by the apparent decrease in masticatory function [88]. In addition, BDNF expression in the hippocampus of mice receiving soft chow was decreased compared to those fed with standard chow, whereas no changes on the BDNF receptor were found in either group [88]. Moreover, the reduced expression of BDNF was consistent with a decrease on the synapses, leading to degraded neuronal structure and therefore neural function [88]. These findings are consistent with those of a recent systematic review of animal studies that identified a relationship between altered mastication and cognitive impairment characterized by decreased expression of BDNF in the hippocampus, decreased synapses, low performance in behavioral evaluations, and diminished memory and spatial location [89••]. Interestingly, male rats fed with standard chow exhibited higher expression of BDNF hippocampus than those fed with either soft or hard chow [90]. This result poses the question of whether reduced and increased masticatory functions are risk factors for cognitive impairment, suggesting a focus on clinical conditions ranging from loss of teeth (and therefore decreased masticatory function) to masticatory muscle parafunction. Short-term exposure of young adult male mice to a soft diet results in dysregulated expression neurodegenerative condition–related genes such as TREM2, DAP12, APOE, and CD33 in the microglia, suggesting that soft diet has an immunomodulatory role as a risk factor for cognitive impairment [91]. Also, mastication on one side only has been shown to affect BDNF gene expression in the hippocampus, with cognitive impairment evaluated using the Morris Water Maze test in young adult male mice [92•]. In addition, using the MWM test, it was determined that reduced physical activity and reduced masticatory function in adult and aged mice affected their memory and learning skills, but these were restored when normal mastication was enabled [93]. The reduction of the branches in the astrocytes of the group with reduced physical activity and masticatory function [93] is intriguing, suggesting the need for more research into the role of the mastication as a neuroprotective musculoskeletal activity. For instance, in humans, masticatory function has been evaluated as a neuroprotective activity based on its clinical correlation with increased brain blood perfusion [27].

Irisin is a myokine released in response to physical activity, downstream of PGC1α/FNDC5 pathway activation, after FNDC5 cleavage [94, 95]. Irisin stimulates BDNF expression in the hippocampus [96], and is believed to mediate the effect of physical activity on BDNF expression [94, 95]. Continuous physical training increases BDNF and Irisin serum levels, with benefits for cognitive performance, measured as the working memory (part of short memory that is a cognitive ability that can hold the information in mind for executing cognitive function tasks [97]) in adults aged 50 to 70 years [98]. In male mice, injection of Irisin to the hippocampus after physical restraint improved the cognitive response to memory tasks. However, female mice did not benefit from Irisin administration, suggesting a sex-dependent effect [99]. Additionally, in FNDC5 knockout mice, absence of Irisin diminished cognitive skills (spatial and learning memory) after voluntary physical activity when compared with wild-type animals [100]. Interestingly, the same study showed that systemically administered Irisin was able to cross the blood–brain barrier and partially rescue cognitive impairment in two AD mouse models [100]. The mechanism of Irisin action remains to be fully understood. However, the use of AD mouse models has revealed a potential role of Irisin in neuroinflammation control [100] and cognition improvement after physical activity, with increased levels of FDNC5, BDNF, and IL-6 [101]. These findings can be compared and contrasted with clinical data about the correlation between Irisin levels in serum [102] or cerebrospinal fluid [103] and neurodegenerative/inflammatory biomarkers in conditions that affect cognition [102, 103].

### Osteokines and Cognition

Osteokines are molecules released by osteoblasts and osteocytes. Among them, the osteoblast-derived protein osteocalcin or bone γ-carboxyglutamic acid (Gla) protein has been proposed to impact cognition [76, 104, 105]. Compared to baseline measurements, serum levels of osteocalcin increase after intense controlled physical activity to a similar extent in women and men [106]. A correlation study in young men exposed to reduced physical activity followed by a single session of high intensity training found that serum levels of both BDNF and undercarboxylated osteocalcin—the hormonally active form of the protein—were increased [107]. Irisin serum levels were also elevated after the intervention [107]. However, whether there is a molecular link among these molecules remains to be determined.

## Musculoskeletal Health and Alzheimer’s Disease, the Potential Connection

Epidemiological evidence indicated a bidirectional relationship between musculoskeletal health and Alzheimer’s disease (AD); however, the shared pathways underlying this relationship are unclear [40]. Beeri et al. (2021) conducted a longitudinal study in the USA to examine the association between sarcopenia and AD incidence [108]. At baseline, 1175 men and women without dementia (mean age = 80.9 years) underwent cognitive testing and assessed sarcopenia parameters each year over a period of 5.6 years. Sarcopenia parameters included muscle mass measured by bioelectrical impedance analysis, muscle function by gait speed, and handgrip strength by a Jamar hydraulic hand dynamometer. Of note, commonly used sarcopenia definitions were not adopted in this study and, instead, cases with sarcopenia were identified using continuous measures of sarcopenia parameters by applying sex-specific binary classifications. Cognitive function was assessed globally (using MMSE and composite scores) and in five specific domains. Clinician-diagnosed dementia cases numbered 243 (78.6% women). This study reported that severe sarcopenia at baseline was associated with a higher risk of incident of AD and a steeper cognitive decline. Among the sarcopenia parameters, poor muscle function and low handgrip strength rather than low lean mass were identified as better risk indicators for AD. This study considered demographic characteristics (e.g., age, sex, and education), seven chronic health conditions (e.g., diabetes, heart diseases, and stroke) and lifestyle factors (e.g., smoking) as confounders; however, no fundamental mechanisms were tested or reported in this study.

Data from the Framingham Offspring Cohort Study in the USA were used to examine the association between BMD and brain structure, and BMD and cognitive function [109]. This study included 1905 men and women (mean age =66 years). BMD of the hip was measured using DXA. Cognitive function was assessed using a series of comprehensive cognitive tests including a neuropsychological battery that included executive function, processing speed, verbal and visual memory, and IQ tests that included dimensions such as abstraction, reasoning, verbal comprehension, and categorization (Wechsler Adult intelligence Scales), and visuo-perceptual skills (Hooper Visual Organization test). Measures of total brain volume, hippocampal volume, and white matter hyperintensity volume were obtained by MRI. This study reported sex-specific associations between higher BMD and better cognitive performance and less white matter hyperintensity burden. The authors proposed cumulative estrogen exposure as the potential underlying mechanism [109]. A large-scale study using a neurobiological approach combining neuroimaging techniques with the biological mechanism is expected to be conducted in this area [44].

Animal models have also been used to determine whether there are associations between cognitive impairments and musculoskeletal deficits. For this, mouse expressing mutations reported in humans with AD have been studied. One of the models is the mouse expressing the Swedish mutation of the amyloid precursor protein, which exhibit bone loss and increased osteoclastogenesis in young but not old mice, suggesting the changes in bone are not explained by the deposit of β-amyloid in the brain, which occurs in old mice [110]. Another model in which both the APP and presinilin1 are mutated (APP/PS1) showed reduced bone mass, but in this case the appearance of the plaque precedes the bone defect [111, 112]. This evidence suggests a disconnection between the central nervous system and the skeletal phenotype. Consistent with this notion, we recently reported a bone and skeletal muscle phenotype in a mouse model in which the R47H variant of the triggering receptor expressed on myeloid cells 2 (TREM2) was globally expressed [113••]. TREM2 R47H is associated with increased risk of AD, frontotemporal dementia, and Parkinson’s disease in humans and mice [114–117]. The changes in musculoskeletal system were present even though the mice expressing TREM2 R47H do not show cognitive deficits and required the presence of additional mutation to increase the appearance of AD-like symptoms [113••]. These pieces of evidence suggest that the mechanisms of bone loss in AD patients might be independent of the central neuropathology

## Future Directions

Future clinical studies could test lifestyle and/or pharmacological interventions that target musculoskeletal parameters associated with cognitive health to identify how particular interventions intended to improve musculoskeletal health in aging people might affect their cognitive status [118]. Further, brain imaging and neuro bio-techniques could be used to investigate the underlying mechanisms for concomitant changes in brain and musculoskeletal health in humans. In addition, studies on the associations between oral health and cognitive impairment are needed in the context of the musculoskeletal deficits, particularly with aging.

## Conclusion

Recognizing the interplay between musculoskeletal deficits and cognitive impairment may have important translational implications, particularly because musculoskeletal health is responsive to behavioral modification. The bi-directional nature of links between musculoskeletal health and cognitive function remains somewhat obscure and their elucidation could be central to informing clinical practice and shaping public health policies for improving/maintaining physical and cognitive health.
